# Treating depression where there are no mental health professionals

**DOI:** 10.2471/BLT.17.030317

**Published:** 2017-03-01

## Abstract

Many people with depression and other mental health problems can be treated successfully by community health workers, but so far no country has scaled up this approach. Vikram Patel talks to Fiona Fleck.

Q: Why did you become interested in mental health?

A: I went into psychiatry at a time when it was an extremely unattractive field and far more stigmatized than it is today. Having ranked at the top of the final university medical examinations in Mumbai, I could have chosen any specialty. At first I was interested in neurology, fascinated by the brain and its mysteries. But during my medical studies it struck me that in neurology the symptoms and signs of the condition were all important. Psychiatry was the only field where the person was central. Also, psychiatry presented a unique bridge between medicine and society.

Q: This year's World Health Day is devoted to addressing depression, can you tell us about the burden and causes of this condition?

A: Depression is probably the most well researched mental disorder globally. A 2013 systematic review for the Global Burden of Disease project identified more than 100 studies from around the world estimating the prevalence of the condition. It found that 4–5% of adults might meet diagnostic criteria for depression, which we could extrapolate to hundreds of millions of people worldwide. The global literature on depression shows that common risk factors associated with depression are related to social disadvantage and deprivation, such as low level of education, job loss, indebtedness, social exclusion or marginalization and violence. Women are also at higher risk of having depression, this is partly related to interpersonal violence and other consequences of gender inequity.

Q: What does that mean for societies?

A: Depression not only affects an individual. In a mother, it affects her child’s growth and development. Depression affects a person’s ability to work, making that person less productive. This not only affects households but the entire economy. A group of economists recently modelled the overall cost of depression for the World Economic Forum and came up with estimates that ran into trillions of dollars. In addition, let’s not forget suicide. While this is classified as a health outcome in its own right and not as a consequence of a mental health problem, many people who attempt suicide are depressed.

Q: Is depression curable? What treatment is needed for this condition?

A: Globally, the strongest evidence is for brief psychological treatments based on cognitive, behavioural and interpersonal mechanisms. We recently conducted a systematic review of studies of psychological treatments delivered by non-specialist providers and identified 27 randomized controlled trials from low- and middle-income countries. Since our review, the findings of at least two major trials have been published, including our own study on the Healthy Activity Program in India in the *Lancet* in January. Most of these trials testify to the effectiveness of these treatments in promoting remission and recovery. There is a smaller evidence base for antidepressants. Both talking treatment and drugs are most effective in people with severe depression. For a significant proportion of people with mild to moderate depressive symptoms, self-care and low-intensity care provided by community members are just as effective as more structured clinical interventions such as psychotherapy.

“What I think does work is disclosure: people coming out and talking about their experience of depression.”

Q: The World Health Day theme “depression: let’s talk” is about seeking help. How can we counter the stigma that often prevents people from seeking help?

A: Stigma is a major challenge and there is no simple solution. A recent review showed limited evidence for many strategies to address stigma. For example, promoting the concept of depression as a biological brain disorder actually led to more negative attitudes, as it suggested that the condition was an immutable aspect of the person’s biology. What I think does work is disclosure: people coming out and talking about their experience of depression. In this respect, the World Health Day message is spot on. In addition, even if it’s hard to change people’s attitudes, we can enforce laws that reduce discrimination, for example removing barriers to education and employment for people with mental health problems. Addressing discrimination is perhaps more valuable and feasible than trying to address stigma. Many people may have a negative attitude to people with mental health problems, but what matters most is that they do not deny them a job or education.

Q: How can we encourage people to seek care for mental health conditions?

A: We recently published a paper in *Lancet Psychiatry* describing a programme in rural India where, over an 18-month period, we brought about a six-fold increase in the proportion of people with depression seeking care. Our approach emphasized a grassroots approach in which local people raised awareness using language understood by the community, avoided reference to depression as a psychiatric problem, discussed issues such as being in debt and domestic violence, championed self-care as a first-level intervention, and used culturally appropriate media such as clips from Bollywood films. We only referred to depression as a biomedical problem when it was severe and clinical interventions were needed. The awareness raising interventions were provided by community-based workers and lay counsellors, meanwhile evidence-based interventions for depression and alcohol-use disorders were made more readily available in both community settings and primary health-care centres.

Q: What do you do in communities without mental health professionals?

A: Empower people for self-care and deploy people in the community to care for others, both with the appropriate training and support. As mentioned, we just completed a review of this approach and found that six to 10 brief treatment sessions of 30 to 40 minutes for patients with severe depression, typically delivered in people’s homes or primary health centres, are effective in promoting remission and recovery. One of the most important findings of this review is that it debunks the myth that patients in developing countries prefer medication to talking treatments. If talking treatments are provided in a contextually sensitive and affordable manner, their acceptability and feasibility is very high.

Q: Has this approach been scaled up?

A: Despite the robust evidence for the acceptability and effectiveness of using community workers to deliver psychosocial interventions, I cannot think of one country or region where this approach has been taken to scale. For example, in India there are many small-scale projects delivering mental health care in places where there are no psychiatrists but what we really need is the full integration of this approach into government health-care systems, for delivery at the primary care level. This is what the PRIME (programme to reduce the treatment gap for mental disorders) consortium funded by the British government, in five low- and middle-income countries, is trying to achieve.

Q: How would the approach work if scaled up?

A: The idea is to train millions of community health workers and people in communities worldwide to deliver evidence-based psychosocial interventions. This approach can not only address mental health problems in low- and middle-income countries, but also in high-income countries where the treatment gap is high in spite of substantial specialist resources. Where mental health professionals are available, they need to provide training, quality assurance and referral pathways for complex mental health problems that do not readily respond to treatment. Digital technologies can play a role in the promotion of self-care, and in training and supervision of community workers.

Q: Why do developed countries rely heavily on medicines to treat depression when talking treatment is highly effective? 

A: Mental health care has become heavily medicalized, dominated by a psychiatric profession in which the prescription of medications is customary. However, people in developed countries are increasingly seeking non-pharmacological options for their recovery, ranging from biomedical psychological treatments to spiritual and traditional approaches, such as yoga. The idea of using lay people in the delivery of mental health care is often resisted by mental health professionals, including clinical psychologists, who argue that it is not safe or effective, in spite of evidence to the contrary. Perhaps they see this as a threat to their professional authority and control over these treatments and health conditions.

Q: How did you start the Sangath nongovernmental mental health organization in Goa? What was new about it?

A: I founded Sangath with six colleagues in 1996. Today it ranks among India’s leading public health research institutions. Sangath started primarily as a centre for children with developmental and mental health problems that later broadened to providing care for all population groups. There was a huge demand for such care and we were inundated with referrals. However, many families could not afford the long-term specialist care and were often unable to come to our facility regularly. So we started delivering care in community and primary-care settings by non-specialist workers and evaluating the effects. Sangath pioneered this approach in collaboration with academic and government partners for a range of different mental health conditions, from autism and alcohol dependence to depression and schizophrenia.

“Mental health is a global public good in and of itself. We must strive to deliver whatever works to those who need it.”

Q: What would you say to governments that assign mental health care low priority?

A: There has to be a fundamental value that we place on mental health. Mental health is a global public good in and of itself. We must strive to deliver whatever works to those who need it: our goal as implementation scientists is to develop effective ways to achieve this goal while maximizing acceptability to patients, their families and those who ultimately have to pay for these services. One thing is for sure: mental health is just as important as physical health, and as with physical health, we cannot deliver mental health care for free.

